# The Diagnosis of Malignant Endocarditis

**Published:** 1899-01-21

**Authors:** F. Percival Mackie

**Affiliations:** Assistant Medical Officer, County Asylum, Shrewsbury; late Assistant House Physician at Bristol General Hospital.


### HOSPITAL CLINICS AND MEDICAL PROGRESS.

### THE DIAGNOSIS OF MALIGNANT ENDOCARDITIS.

By F. PERCIVAL MACKIE, L.R.C.P.Lond., M.R.C.S.Eng., Assistant Medical Officer, County Asylum, Shrewsbury; late Assistant House Physician at Bristol General Hospital.

(*Continued from page* 260.)

THIS so-called typhoid type is said to be commoner after pneumonia than is the pyæmic type, and is characterised by marked somnolence and prostration, which occurs early, and comparative infrequency of rigors or sweats. The addition of splenic enlargements, tympanites, and petechial rashes, and sometimes diarrhœa, afford still more justification for the term typhoid as applied to this type.

When the disease follows a suppurating wound, puerperal fever, erysipelas, and other allied conditions, it is apt to assume a sthenic form, the so-called pyæmic type. Such a condition is more likely to occur if the valves have been previously damaged by disease or strain. This is only clinical proof of the experimental evidence which shows that pyogenic organisms injected into the blood stream in rabbits showed no special tendency to affect the heart valves till these were artificially injured, when ulcerative endocarditis usually followed. Thus we may argue that, although acute rheumatism cannot claim to be the direct antecedent of malignant in anything like the same proportion that it is of simple endocarditis, yet we must admit that past attacks of rheumatic fever undoubtedly leave the valves in a condition more susceptible to parasitic invasion than are healthy ones. The chief characteristic of the septic or pyæmic type is the frequency of rigors and drenching sweats, and also the great irregularity of the fever, which may vary to the extent of six or eight degrees between morning and evening. The presence of rashes is not uncommon in this or any of the types, and the following are the commonest: (1) Diffuse erythematous rashes like the prodromal rashes of certain fevers. (2) Petechial rashes, with subcutaneous hæmorrhages of larger size, which occur only in the most severe cases, generally of the typhoid type. (3) Diffuse staining extravasation of dark blood under the skin on sites exposed to pressure, *e.g.*, heels and buttocks, occurs in nearly all cases shortly before death. (4) Papular rashes, due to multiple emboli in the skin. These only occur in a few cases, and generally indicate the bursting of an endocardial abscess. If the patient lives the papules go on to suppuration, *i.e.*, they become pustules.

Stress is sometimes laid upon the difficulty of diagnosing between the septic type of endocarditis and pyæmia, but it should be realised that the diseases are practically one and the same. The only difference is that the source of the septic material in one case is the endocardium, and in the other a cellulitis or what not, or, to put it another way, suppurative endocarditis may be looked upon as a pyæmia, in which the endocardium has become affected instead of a joint, the pleura, or pericardium. The last case will be given rather more in detail, as it is a good example of the composite nature of the disease in that it presents some symptoms of almost all types, and also because of its great severity and extremely rapid course.

S. S. C., æt. 22, labourer, admitted to Bristol General Hospital, said to be suffering from purpura hæmorrhagica. History showed that he had had rheumatic fever three years ago. He had been suffering from winter cough, spitting phlegm, and night sweats for several winters past, but more recently he had been quite well. He had been taken ill seven days before admission with a rigor, pains in limbs, headache, and vomiting. Pains in the limbs got worse, vomiting was persistent and without relation to food, and severe spinal pain came on latterly. Eyesight was failing rapidly. He complained also of pain across the chest and palpitation. He had diarrhœa and blood in the stools, blood in the urine, epistaxis, some petechiæ over abdomen, and also abdominal tenderness and fullness. He was just conscious on admission, but in a few hours sank into coma. On examination his temperature was 103. Pulse 120. Sordes on teeth and lips. Rigidity about neck, arms, and legs. Purpuric spots on legs and trunk and subcutaneous hæmorrhages under heels and over sacrum. Heart apex beat half an inch outside nipple in fifth intercostal space. Soft aortic and mitral systolic murmurs, also a soft pulmonary systolic. Lungs showed soft friction sounds over left base, and some harsh almost tubular breathing in patches. Some fine friction sounds scattered over the upper parts of the lung. Urine contained blood and some albumen, no indican, Erlich's reaction negative; no tubercles seen on choroid, but marked optic neuritis. In addition he had tympanites, enlarged spleen, and diarrhœa. An occasional rigor drove the temperature up to 106. A few hours before death a large crop of pustules appeared over abdomen and thighs, feeling like small shot. The patient died eight days from onset, an unusually short time even for very acute cases. The post-mortem showed extensive ulcerative change in the aortic valves, and a recently-ruptured abscess was seen as a ragged, cup-like cavity, which had occasioned the shower of emboli seen in the kidneys and intestines, and which had given rise to the phenomena of the rash referred to. Permission was not given to examine the head, but undoubtedly there was meningitis present. This case shows well the difficulties that may arise in coming to a conclusion in a case of this sort. Thus, here there were reasonably good grounds for diagonising—(*a*) General tuberculosis, (*b*) pyæmia, (*c*) typhoid fever, (*d*) ulcerative endocarditis, (*e*) cerebro-spinal meningitis, and possibly pneumonia with meningeal complications and purpura hæmorrhagica.

Besides the ordinary signs peculiar or common to the various diseases from which ulcerative endocarditis has to be diagnosed, stress may be laid upon the value of the careful examination of the blood: (1) Widal's method, by which for practical purposes we can prove or exclude the presence of enteric fever. (2) Leucocytosis, which is absent in general tuberculosis, typhoid fever, and malaria, of those diseases which concern us, and is present to a marked degree in all the others. (3) The presence of micro-organisms in the blood, as shown by cultivation. Organisms can generally be obtained in pyæmia and malignant endocarditis, and occasionally in purpura hæmorrhagica, and are absent in pneumonia, general tuberculosis, and typhoid. Malarial organisms are found in ague (with which the disease has been confounded). The examination of the sputum or other morbid product will, of course, be additional proof or otherwise. Thus, here the finding of tubercle bacilli or pneumococci in the sputum would have pointed to the lung disease as being primary, and the presence of pyogenic cocci would suggest that the lung mischief was secondary to some disease elsewhere.

To summarise, the examination of the blood may go a good long way toward the diagnosis of the various diseases simulating endocarditis, the one from the other. Thus, in this case, the absence of Widal's reaction excluded typhoid fever; the presence of marked leucocytosis, and also the discovery of large numbers of staphylococcus pyogenes aureus in the blood proved that it was neither general tuberculosis, typhoid, nor ague, and probably not cerebro-spinal meningitis (in which latter the organisms are confined to the membranes). It therefore appeared probable that it was either pyæmia or endocarditis, and the marked signs of heart disease which indicated the latter proved correct.

There are several other diseases with which malignant endocarditis has been or may be confounded, but the chief have been indicated; and after the clinical history, symptoms, and signs have been carefully weighed, and the valuable evidence that bacteriology and microscopy place at our disposal has been brought to bear on the case, there will be fewer cases of malignant endocarditis that will escape detection.

